# Divisions of general practice in Australia: how do they measure up in the international context?

**DOI:** 10.1186/1743-8462-4-15

**Published:** 2007-07-13

**Authors:** Judith Smith, Beverly Sibthorpe

**Affiliations:** 1 Health Services Management Centre, School of Public Policy, University of Birmingham, 40 Edgbaston Park Road, Birmingham, B15 2RT, UK; 2 Australian Primary Health Care Research Institute, Building 62, Australian National University, Canberra, ACT 0200, Australia

## Abstract

**Background:**

Since the late 1980s, there has been evidence of an international trend towards more organised primary care. This has taken a number of forms including the emergence of primary care organisations. Underpinning such developments is an inherent belief in evidence that suggests that well-developed primary care is associated with improved health outcomes and greater cost-effectiveness within health systems. In Australia, primary care organisations have emerged as divisions of general practice. These are professionally-led, regionally-based, and largely government-funded voluntary associations of general practitioners that seek to co-ordinate local primary care services, and improve the quality of care and health outcomes for local communities.

**Discussion:**

In this paper, we examine and debate the development of divisions in the international context, using six roles of primary care organisations outlined in published research. The six roles that are used as the basis for the critique are the ability of primary care organisations to: improve health outcomes; manage demand and control costs; engage primary care physicians; enable greater integration of health services; develop more accessible services in community and primary care settings; and enable greater scrutiny and assurance of quality of primary care services.

**Summary:**

We conclude that there has been an evolutionary approach to divisions' development and they now appear embedded as geographically-based planning and development organisations within the Australian primary health care system. The Australian Government has to date been cautious in its approach to intervention in divisions' direction and performance. However, options for the next phase include: making greater use of contracts between government and divisions; introducing and extending proposed national quality targets for divisions, linked with financial or other incentives for performance; government sub-contracting with state-based organisations to act as purchasers of care; pursuing a fund-holding approach within divisions; and developing divisions as a form of health maintenance organisation. The challenge for the Australian Government, should it wish to see divisions' role expand, is to find mechanisms to enable this without compromising the relatively strong GP engagement that increasingly distinguishes divisions of general practice within the international experience of primary care organisations.

## Background

Since the late 1980s, there has been evidence of an international trend towards more organised or managed primary care [1-4]. This has taken a number of forms including: the emergence of primary care organisations (PCOs); the use by governments of primary care as the arena for implementing a range of health care reforms aimed at achieving specific public objectives; a policy intent in relation to shifting services away from hospital settings and into the community; and an overall raising of the profile of primary care within wider health systems. Underpinning such developments is an inherent belief in the evidence that suggests that well-developed primary care is associated with improved health outcomes and greater cost-effectiveness within health systems [5].

PCOs have been defined as 'bodies [seeking] to increase the influence of primary care professionals, and in particular general practitioners (GPs), in health planning and resource allocation, and in the health system more generally' [[2], p1]. They are based on a fundamental belief that there is value in having GPs closely involved in the leadership of organisations within health care, and that this 'clinical engagement' will in itself facilitate the progress made by local organisations [6]. They provide management and organisational support to general practice and analysis of the international experience of PCO development suggests that core roles for these new organisations are as follows [2]:

- to improve health outcomes;

- to manage demand and control costs;

- to engage primary care physicians;

- to enable greater integration of health services;

- to develop more accessible services in community and primary care settings; and

- to enable greater scrutiny and assurance of the quality of primary care services.

Primary medical care in Australia is provided mainly by independent general practitioners on a fee-for-service basis predominantly funded by the Australian Government through Medicare, under which all Australians are entitled to a 100% rebate of the fee listed on the Medicare Benefit Schedule. Some 80% of services are bulk-billed, that is the doctor accepts a schedule fee as full payment. For the remaining 20% of services, patients make a co-payment which is at the discretion of the provider. There is no system of patient registration with a GP or practice, underlining the policy emphasis on a business model of general practice where patients are free to consult any GP. Commencing in the early 1990s there have been a number of changes to general practice organisation and financing, partly in response to concerns about rising costs, a likely consequence of a fee-for-service system that research evidence tells us is likely to encourage practitioners to increase the quantity of care provided in order to maximise income [7]. Other concerns relate to variations in the quality of and access to general practice, and difficulties in recruiting and retaining primary care professionals in many parts of Australia. Reforms that have been introduced to address these concerns include: a practice incentive programme to improve quality and accountability of GP services such as paying for immunisations and prescribing reviews; a rural incentives programme that includes paying GPs to relocate to and stay in rural and remote communities and outer urban areas; and amendments to the fee schedule that encourage participation in care planning and case conferencing and more multidisciplinary care [2].

The major structural reform has been the creation of divisions of general practice. These are professionally-led and regionally-based voluntary associations of GPs that seek to co-ordinate local primary care services, and improve the quality of care and health outcomes for local communities. The first divisions were established in 1992 and there are now 119 across Australia, ranging in size from eight to over 600 GPs, with 94% of GPs being a member of a division [8]. Divisions were originally established as general practitioner organisations, although over time, there has been a growing contribution by nurses and other health professionals to the work programmes of divisions. In this paper, however, we focus largely on divisions as GP membership organisations when exploring their experience and potential future. There are state-based organisations to represent and co-ordinate divisions at a state/territory level, and a national co-ordinating body, the Australian General Practice Network (AGPN) which was, until 2006, known as the Australian Divisions of General Practice. Together these form what is known as the divisions' network.

In this paper, we argue that divisions are part of the international PCO 'movement' and use a published framework about the roles of PCOs to debate how divisions might develop in the future. We recognise the contestable nature of this approach, however we believe that international research evidence on PCOs presents a useful basis upon which to analyse the policy options available to divisions of general practice in Australia. Thus we use the six PCO roles identified above to critique this particular manifestation of managed or organised primary care. We go on to consider how divisions 'measure up' internationally and what opportunities exist for future development.

## Discussion

### Establishment and development

Divisions were originally borne out of an idea from general practice that was picked up by the Australian Government. This is in contrast to PCOs in New Zealand and England which were, in their initial stages, largely formed in response to a perceived threat to general practice from government (as with the independent practitioner associations in New Zealand), or as a way of 'gaining strength in numbers' (as experienced by GP fund-holders and GP commissioners in the 1990s in England) [1,2]. Divisions' funding is allocated on the basis that it is to be used by divisions to support GPs to improve the quality of general practice care and health outcomes for communities. Unlike the current English primary care trusts (PCTs) or New Zealand primary health organisations (PHOs), divisions do not focus on services beyond general practice. Some elements of primary health care are delivered by state/territory agencies, and medical specialists, who provide ambulatory services and yet are not members of divisions.

Within the broad parameters outlined for divisions, no clear direction was initially articulated by Government and over the period 1992–2002, they were largely left to organise themselves and determine local priorities for general practice support and development. This can be seen on the one hand as a sensible and pragmatic approach to facilitating the development of GP groupings in a context that was firmly rooted in the private business/independent model of general practice, recognising that it could be counter-productive for the government to be seen to be dictating terms and trying to contract with GPs outside the usual arrangements for agreeing practice fees, something that would be unthinkable in the Australian context. On the other hand, it could be viewed as a missed opportunity in relation to shaping divisions in accordance with federal and state health priorities.

In 2002, the Australian Government announced a review of the role of divisions which reported in 2003 (the Phillips Review) and not surprisingly found great variation in approach, service development and allocation of funding [9].

'Some Divisions are almost totally consumed by their role of helping general practitioners with their businesses and patients; others, to various degrees, have progressed to addressing the broader primary health care needs of their communities....The entire Divisions network should play a stronger and more consistent role in primary health care.' [[9], p6]

The review concluded that divisions needed better defined goals, clarity of roles, increased accountability for outcomes and taxpayers' funding, improved consistency of performance and governance across the network, greater alignment with territory and state boundaries and an increased focus on the delivery of services [9]. This review was an important policy signal about the Australian Government seeking to regain the initiative in relation to divisions. It was also an indication that there was a move towards wanting greater clarity about what the government was getting in return for its funding of divisions. This reflected an international trend towards more organised and managed primary care whereby traditionally independent and autonomous general practice is expected to work in a more collegial and corporate manner towards public and primary health goals as agreed at a local and national health system level [2].

The Australian Government responded to the Phillips review in April 2004 [10] and articulated what it considered to be the future core roles for divisions, namely that they would be able to:

- support GPs and practices with a changing primary care environment;

- improve access;

- encourage integration and multi-disciplinary care;

- focus on prevention and early intervention;

- better manage chronic conditions;

- support quality and evidence-based care; and

- ensure a growing consumer focus.

The Government also confirmed a further round of funding and assured the network that it would support work to improve primary care, provide new long-term opportunities for divisions, and promote and support a culture of high performance, good governance and accountability. In return for this, it signaled its intention to 'get more serious' in relation to the performance of divisions, and set out ways in which it would reward high-performing divisions, deal with under-performing divisions, and seek to merge divisions lacking 'critical mass' [10]. This heralded the implementation of a new, national quality framework for divisions that would have been unthinkable even five years previously.

Divisions have, in comparison with international experience, been extremely effective in retaining almost exclusive GP leadership. In the UK and New Zealand, PCOs have been used latterly by the national health system as a route for managing and shaping primary care and community services, with previously GP-run and led organisations being to some degree (or in England to a great degree) 'taken over' by the state and subject to national direction and objectives [11]. There is tentative evidence that this absorbing of professionally-led PCOs into the wider system of health management may compromise both GP engagement and the associated work to improve the quality of primary care services through peer review [12-14].

Given what is known from research about the importance of doctors' engagement with and involvement in health care organisations [6], divisions in Australia might wish to heed the experience of their colleagues in New Zealand and England, ensuring that they can remain at arm's length from government control. Retaining GP ownership and governance of divisions and negotiating contractual arrangements with national and state government about what divisions will deliver in return for infrastructure and other targeted funding would seem to be an evidence-based strategic approach [1,11-13]. To compromise the strong and sustained GP leadership and control enjoyed by divisions would, on the basis of international research evidence, threaten the unique strength of PCOs – the engagement of primary care physicians in collective action to improve and extend primary care services and infrastructure.

In Australia, co-ordinating structures – the state based organisations and AGPN for divisions – were established and funded by the Australian Government. In this respect, divisions differ from the independent practitioner association (IPA) movement in New Zealand (with the IPA Council), the community governed sector in New Zealand (with Healthcare Aotearoa), and GP fund-holding and GP commissioning in the UK (with the National Association of Primary Care and the NHS Alliance respectively), where the co-ordinating organisations were set up and funded by PCO members.

Whereas in Australia, the state-based organisations and AGPN remain the primary co-ordinating bodies for PCOs at state and national level, in New Zealand and England, government has intervened to develop formal co-ordination, funding and governance arrangements for PCOs that have, as we have noted earlier, 'taken over' or sought to manage and influence much more strongly, pre-existing GP-led organisations. The AGPN, together with the state-based organisations, has taken the lead in articulating and developing the role of divisions, encouraging them to widen their remit and share good practice, while also providing them with management support and creating a 'sense' [15] of what divisions are and can be to the wider world. However, the lack of contractual obligations between AGPN and divisions, and divisions and their members is a key barrier to merging or exploiting each level and ultimately the Australian Government exercising more influence on the activities of divisions.

Using the six roles for PCOs identified by Smith and Goodwin [2] we now examine the experience of divisions within the international 'PCO movement'.

#### 1) Improving health outcomes

Establishing the extent to which PCOs have been able to improve health outcomes is notoriously difficult, given the challenges inherent in attributing any observed changes to a particular organisational form as against other causal factors [1,2]. Nevertheless, evaluation of the implementation of PCOs has shown a tendency to start to focus on population health improvement once initial attention to organisational development and practice-based primary care services has been embedded [2,13,14]. Where PCOs are able to contract with practices and other providers against specified performance and outcomes frameworks, there is evidence that providers can achieve specified health outcomes. For example, the UK's new general medical services contract implemented in 2004 has enabled the measurement and assurance of outcomes in areas such as immunisations and vaccinations, the regular monitoring of chronic disease states, the cessation of smoking, and the achievement of health screening targets [16].

The extent to which PCOs truly adopt a population health perspective and focus on health improvement (as opposed to general practice service development) remains open to question. However, research highlights the practical and cultural barriers that need to be overcome if general practice-led organisations are to adopt a population health approach that addresses the wider determinants of health [17,18].

In Australia, the explicit intention of federal government funding of divisions is to bring about improved health outcomes for the populations that they serve. This is reflected in the core roles for divisions [10] – for example, better managing care for people with chronic conditions, and focusing on prevention and early intervention. Divisions are the only Australian health institution below state/territory level that has a geographic and population focus. Until now, it has not however been possible to assess the extent of divisions' impact on population health outcomes in any consistent way. This has changed with the National Quality and Performance System (NQPS) for divisions which includes outcome indicators in the three chronic disease domains of diabetes, mental health and asthma.

The major challenge facing divisions is the extent to which they can use their population focus to influence the nature and quality of care delivered by their member GPs and practices. This was repeatedly raised during the national consultations undertaken during the development of the NQPS indicators when many commentators argued that divisions' influence was limited and that the quality of their performance should therefore not be assessed in terms of population health outcomes. If the explicit intention of divisions' funding is improved health outcomes this argument is highly problematic.

On the ground meanwhile, divisions are increasingly adopting a more population health focus. For example, the Annual Survey of Divisions 2004–2005 noted that 99% divisions provided immunisation programmes or activities, 73% divisions provided prevention for type II diabetes, and 42% reported having prevention and early intervention programmes specifically aimed at Indigenous Australians, including nutrition, type II diabetes prevention and immunisation activities [17]. This suggested that, in line with international research evidence on PCOs, divisions were increasingly focusing on population health, as well as primary care development as they moved beyond their initial priorities. The relative organisational stability of divisions, together with their geographical base, mean that these organisations are, in theory, well placed to carry out assessment of the impact of new population health initiatives upon people's health. This is something that has been impossible in other countries, such as England, where organisational restructuring of PCOs has prevented long-term assessment of health outcomes (although the new general medical services contract now enables practice-level assessment of outcome indicators, irrespective of wider PCO reorganisations).

#### 2) Managing demand and controlling costs

Assuming a role in managing demand for health services elsewhere in the health system and controlling costs, has not to date been a core function of divisions. Some limited fund-holding is taking place through initiatives such as 'More Allied Health Services' and 'Better Outcomes in Mental Health Care', where divisions hold budgets for and develop local services. This is designed to increase patient access within existing Medicare funding arrangements, rather than try new funding models to help manage demand or control costs. It would seem that divisions are now well placed to extend their role in fund-holding and managing care for defined populations. Extensive international research evidence exists in this area, and that evidence would suggest that fund-holding divisions should be able to make an impact on the delivery of primary and intermediate care, and that the relative stability of the policy context enjoyed by divisions over the past 15 years puts them in a good position to monitor outcomes that result from such activity [12].

There is strong interest among some divisions in assuming budgets. Led by AGPN, some divisions have proposed that they hold ongoing, population-based budgets for defined packages of care (e.g. prescribing, aged care, after-hours care, long-term conditions). The Australian Government has however to date been reluctant to create budgets to devolve to divisions. This is despite evidence from its own co-ordinated care trials carried out in the 1990s (trials that involved primary care budget holding by groups of professionals charged with managing care for people with chronic conditions and complex needs), that concluded that fund-holding had the potential to improve patient outcomes if it was implemented with appropriate incentives for professionals, prior community debate, and careful monitoring and evaluation [20]. Reasons for this reluctance on the part of government might include: research evidence that suggests that primary care-based purchasing has higher transaction costs than larger area funders [1,12]; the complexities inherent in pooling resources across different levels of government (national and state/territory); the power of the Australian medical establishment and its traditional opposition to fund-holding on the basis that it draws doctors into health care rationing; manifest unevenness in capacity across the divisions network to take on such a role; and reticence on the part of federal government to allocate large sums of public money to organisations made up of private sector GPs with whom they have no contractual relationship.

It is interesting to note that in New Zealand and England, Labour governments have placed their faith in a collective model of PCO (the PHO and the PCT respectively) as a way of planning and developing population health within a primary care setting. In Australia, with a Liberal (Conservative) government, it would appear that commitment to the independent business model of general practice remains fundamentally more important than further development of collectives (divisions) as a route for population health planning and development.

#### 3) Engaging primary care physicians

International research underlines the importance of effective clinician engagement (especially of GPs) in the work of high-performing PCOs [1-3,12,20,21]. Divisions are recognised as having a focus on engaging primary care physicians in their governance, leadership and activities [9]. The fact that divisions have been able to develop in an incremental and organic manner over more than a decade is an important enabler of GP engagement. However, despite high overall levels of GP membership of divisions, there is concern among them about the number of rank and file GPs who, whilst officially members of divisions, are not actively engaged with the objectives and activities of their local division. This is a problem for the divisions' network if it wishes to promote itself to federal and state/territory government, and to the community, as a reliable platform for primary health care reform. It is also problematic if the quality of divisions' performance rests on what could be achieved through more active member engagement, although the very act of extending the responsibilities and remit of divisions might facilitate this improved involvement by doctors. It is possible that the Australian Government's, and even divisions' ambitions for the network are so far removed from those of some of the GP rank and file that better engagement cannot be achieved without the introduction of much stronger incentives.

Determining what is actually meant by 'GP engagement' in PCOs has been the subject of debate within research, and a study of PCOs in England [22] suggested that grassroots GP involvement in PCO activity could be explored within the following categories:

- attendance at PCO board meetings;

- participation in PCO board sub-groups;

- involvement in GP forums and locality meetings;

- attendance at PCO awaydays;

- provision of comments on PCO discussion documents; and

- participation in PCO education events.

More recent experience of PCTs in England suggests that GP engagement for at least some doctors extends beyond the above activities to include: leading the process of local clinical governance and peer review among practitioners; taking a lead role for a specific clinical area on behalf of the local PCT and its GPs; being a member of the PCT's professional executive committee; and taking responsibility for the redesign of local primary and intermediate care services [23]. Evidence from Australia is that many grassroots GPs have been enthusiastic in their participation in divisions' education events and in attending GP forums and local meetings. However, more extensive engagement in activities such as service development sub-groups, and leading programmes of work appears to have been more patchy, both within and across divisions [9]. In the international context, the particular opportunity presented to Australia's divisions is their longevity as organisations and the fact that they have been able to continue as GP-led and organised bodies, escaping the 'corporatisation' of being absorbed into mainstream healthcare management, something that has befallen English PCTs and arguably, New Zealand PHOs [11,24]. Divisions are therefore well-placed to continue to build on their existing (albeit variable) GP engagement and to find new ways of incentivising GPs (and other primary care professionals) to participate in programmes of service development and health improvement. Achieving this will require careful attention to the design of an appropriate blend of incentives for GPs, developed in such a way that corporate divisional and individual practice needs can be met, for international evidence underlines the critical nature of sustaining GP engagement and avoiding the risks associated with a state 'take-over' of PCOs [11,24].

#### 4) Enabling greater integration of health services

Research evidence highlights the potential for PCOs to use a combination of primary care clinician leadership (including medical, nursing and other professional colleagues), knowledge of local services, and responsibility for some elements of health resource, in order to bring about better integration of services at the interface between primary (community-based) and secondary (hospital-based) care [2,12,13,25]. In Australia, policy makers have increasingly emphasised the role and potential of divisions in this regard [10], particularly in relation to developing better integrated multi-disciplinary care for people with chronic conditions. Indeed, there is evidence that divisions are seeking to integrate health services in a number of ways: across practices within divisions; between divisions and community health services; and between divisions and hospitals [8]. Examples include structured shared care programmes between GPs and specialists in mental health, antenatal, diabetes and aged care; programmes to improve GP-hospital interactions including admission and discharge communication, negotiated discharge planning and GP hospital liaison; and programmes that involve enhanced integration with community-based providers including Quality Use of Medicines, care planning, case conferencing and improved access to mental health services.

The potential for primary care-led organisations to play a role in developing better integrated care is typically seen through the lens of managed care from the United States. Within managed care organisations, there is usually a capitated budget for providing care for a specific group of patients, with an expectation that this will be arranged by the organisation in the most cost-effective and integrated manner [2]. Taken alongside evidence from the UK of the effectiveness of primary care-led purchasing in bringing about improvements in services at the primary-secondary care interface [12], this would suggest that Australian divisions could further improve integration through budget-holding for specified packages of care. As noted above, the chief impediment currently to them taking on this role is government reticence to respond to divisions' proposals for such initiatives.

#### 5) Developing more accessible services in community and primary care settings

The development of an extended range of more accessible primary and community services was a driving force in the setting up of divisions of general practice in Australia [9], independent practitioner associations in New Zealand [26], community health organisations in New Zealand [27], fund-holding and GP commissioning groups in the UK [1,2] and primary care groups and trusts in England [2,25]. Each of these countries has shared a desire to strengthen the provision of services in primary care settings and to use this stronger primary care as a basis for improving population health and developing alternatives to hospital admission. This is the area of PCO activity that has the strongest evidence base in respect of ability on the part of the PCO to bring about change, probably on account of the interest of GPs and other primary care staff in putting time and effort into developing services of direct relevance to their patients, hence resolving what are often long-standing frustrations for GPs and their teams seeking to deliver well co-ordinated and accessible care [12].

In Australia, divisions have undertaken a range of activities to improve access to GP services and to other community-based providers. The former has been predominantly through after-hours care, locum services, residential aged care facilities and in some areas, Aboriginal Community Controlled Health Services. The residential aged care activities have been largely in response to additional Australian Government funding made available to divisions to establish Aged Care Panels to enhance primary medical care for residential clients. Divisions have also been very active in supporting access to other community-based providers including psychologists, counsellors and mental health nurses, dieticians, diabetes and asthma educators, podiatrists, social workers and physiotherapists. Some of this has been funded through the Australian Government's More Allied Health Services programme and some through other sources, including state/territory government initiatives, and divisional core funding.

Through these activities, divisions have acted not only as organisations focused on improving local services but also as support and development resources to individual practices facing challenges related to staffing and capacity. International experience points to the importance of PCOs retaining this focus on practice support and primary care development, even as the PCO might extend its reach into broader population health work and/or more extensive fund-holding [2,25]. The reason for this is that primary care and practice development is crucial to securing and maintaining GP engagement in the work of the PCO which, as we have noted earlier, is a critical success factor for PCOs.

#### 6) Enabling greater scrutiny and assurance of quality of primary care services

PCOs consistently report that one of their main achievements has been to develop a context and framework for quality improvement in primary care, with the existence of a forum for peer review within and across practices being considered of particular importance [1,2,9,13,25,26]. In some cases, this is conceptualised as the development of 'clinical governance' within primary care [11,25] and in others as 'developing evidence-based care' [9]. Examples of what this quality improvement work typically entails include: strategies for reviewing and improving prescribing practice; setting and monitoring standards for areas of chronic disease management within practices; educational activities in support of agreed primary care development plans in the PCO; and incentive schemes for achieving PCO targets in specific disease management areas.

Many divisions have encouraged their practices to engage in accreditation through Australia's independent national accreditation bodies and they have been very active in providing continuing professional development aimed at improving the quality of general practice care. However, a wider role in terms of quality assurance has been more limited. There are a number of divisions that have diabetes programmes that involve the transfer of clinical data from practices to division, and the collation, analysis, interpretation and feeding back of that data to GPs/practices, including anonymised cross-practice comparisons. This type of activity is set to expand with the introduction of the NQPS which provides an incentive to divisions to undertake regional collation and review of clinical data. However, there is a level of resistance among some rank and file GPs to the notion that divisions have a role to play in monitoring the quality of clinical care. Similarly, the Australian Medical Association has remained reluctant to embrace the notion of a quality improvement process at a system level within general practice, fearing some loss of control of the process (and hence of clinical care itself) to government. In a world of greater demands for accountability from both governments and consumers, this is problematic. Interestingly, no entity currently has responsibility for this role in Australia. Medicare investigates over-servicing and other irregularities such as prescribing rates, but clinical data from general practice are not routinely collected and monitored for quality. With much-needed improvements in the information platform in general practice hoping to be realised in the near future, divisions could play an important role in this area. Many would see this as counter to their role in providing GP/general practice support, though the experience of the regional diabetes programmes [e.g. [28]] and primary care collaboratives [e.g. [29]] demonstrates that this work can be done in a way that is about quality improvement rather than scrutiny *per se*.

The experience of PCOs such as IPAs in New Zealand and primary care groups in England, has demonstrated the value of 'effective resource management', namely GPs gathering in professional peer groups to consider and analyse prescribing data [11]. What is clear is that professionally-led groupings of GPs present a forum for peer review and development than transcends traditional practice boundaries and bears witness to a new degree of collegiality and collaborative working in general practice. There has been strong interest among some divisions to implement such programmes, although government appears to be unwilling to agree to arrangements that would allow division-government sharing of the financial benefits of better prescribing, in part needed by divisions to fund the programmes.

## Summary

Established in the early 1990s, there has been an evolutionary and 'bottom up' approach to divisions' development and they now appear firmly embedded as geographically based planning and development organisations within the Australian primary health care system. A more formal set of national objectives has been introduced for divisions, along with a quality and performance framework that reaches into the clinical care provided by member practices. This will allow nationally consistent assessment of divisions' ability to improve health outcomes for the first time.

Divisions have patiently nurtured the involvement of primary care physicians over the past 15 years to achieve almost universal membership, although there is ongoing concern within the divisions' network about the extent of real engagement in divisions of 'rank and file' GPs. Divisions have been active in the areas of service integration and making services more accessible, but they have been afforded almost no role in managing demand or controlling costs and have had little role in directly assuring the quality of primary care services.

Led by AGPN and its state-based affiliates, some divisions have been agitating for a greater role in health planning and purchasing – being given a 'real job' – but this is being resisted by government. There are interesting comparisons to be drawn with the experience of central-local dynamics in New Zealand and England. In both of these latter cases, the government can be said to have sought to 'take over' PCOs to a greater or lesser extent, wanting to use what it perceives is good about them (a more collective approach to general practice, better population health information, a vehicle for peer review and clinical governance, an organisation within which to manage more effectively primary and community services) as a means of achieving wider governmental aims such as reducing health inequalities, improving access to care, developing more integrated services, and controlling demand for secondary care. In New Zealand and England, there are signs that GP engagement has suffered as a result of the increasingly central control of PCOs, with a resulting threat to the effectiveness of PCOs in terms of their original purpose [11].

The challenge for Australia is to negotiate a stronger national and state/territory focus and role for divisions (from the perspective of government and policy makers), whilst resisting the temptation to manage, control and govern divisions and their representative bodies. In particular, our analysis suggests that taking on a greater degree of budgetary management, extending work focused on service integration, exploiting the geographical/population focus of divisions, and the assessment and development of quality in primary care are areas ripe for more attention in the next phase of development of divisions of general practice. This raises the question of what mechanisms could be used by government to ensure that pooled public monies are well-spent in a way that improves public health. Perhaps divisions could assume a role of 'commissioner' [30] of local primary health care services, assessing needs, designing appropriate care, and then actually or notionally purchasing services to meet assessed needs (from practices and other local providers), prior to reviewing what is needed for the next phase.

Options for bringing about this next stage of development of divisions include: making greater use of contracts between government and divisions and between divisions and practices or practice groups; introducing the proposed targets and extending the patient care and clinical outcomes indicators in the NQPS, linked with financial or other incentives for performance; the Australian Government sub-contracting with state-based organisations to act as health plan managers/purchasers of care on behalf of state or federal government; pursuing a fund-holding/GP commissioning approach within divisions, perhaps focused on long-term and chronic conditions; and developing divisions as a form of health maintenance organisation whereby patients sign up with a division to be responsible for providing (and/or buying in) integrated care. In order to take on an expanded role in a publicly funded health system, divisions would need much greater involvement of consumers and other members of the primary health team in their governance, planning and management, consistent with trends in international PCO development [1,2,13]. It may at first sight appear contradictory to assert that divisions should both retain effective GP involvement and also seek to engage consumers and others in the primary health care team in PCO governance and organisation. However, strong engagement of GPs, nurses, other primary care professionals and the public will all be necessary if divisions are to operate as fully functioning PCOs that can both engage professionals in developing primary care services and also account properly to the public and communities they serve.

Divisions might, until a few years ago, have appeared to be relatively late 'adopters' [31] within the broader international experience of developing PCOs. However, there is now evidence that patient and sustained development of divisions within a relatively stable management and policy environment is paying dividends in respect of some GPs' enthusiasm for playing an important role in developing primary health care and general practice more generally within Australia. Divisions appear to have successfully retained a significant degree of clinician leadership and control and now seem to be clamouring for greater responsibility and influence over primary care service development. The challenge for the Australian Government is to find levers and mechanisms to enable this expansion of role to occur without compromising the clinician engagement that, by international comparisons, continues to define divisions of general practice in Australia.

## Abbreviations

AGPN Australian General Practice Network

GP General practitioner

IPA Independent practitioner association

NQPS National Quality and Performance System

PCO Primary care organisation

PHO Primary health organisation

PCT Primary care trust

UK United Kingdom

## Competing interests

Both authors have been involved in health policy development and evaluation in the UK and New Zealand (JSm) and Australia and New Zealand (BS) as researchers and analysts over recent years, including work with and for the English Department of Health, the Australian Commonwealth Government, the New Zealand Ministry of Health, and other research funding bodies.

## Authors' contributions

JSm and BS developed the idea for the paper during a visiting fellowship spent by JSm at the Australian Primary Health Care Research Institute in 2005. JSm prepared the first draft of the paper and BS commented on this and provided supplementary material for subsequent drafts. Both authors read and approved the final draft.
